# Assessing the Safety of Pesticides in Food: How Current Regulations Protect Human Health

**DOI:** 10.1093/advances/nmy061

**Published:** 2019-01-21

**Authors:** William R Reeves, Michelle K McGuire, Milton Stokes, John L Vicini

**Affiliations:** 1 Agricultural Affairs and Sustainability, Bayer U.S. Crop Science Division, Chesterfield, MO; 2 Regulatory Policy and Scientific Affairs, Monsanto Company, Chesterfield, MO; 3 Margaret Ritchie School of Family and Consumer Sciences, University of Idaho, Moscow, ID; 4 School of Biological Sciences and Paul G. Allen School for Global Animal Health, Washington State University, Pullman, WA; 5 Agricultural Affairs and Sustainability, Bayer U.S. Crop Science Division, Creve Coeur, MO; 6 Corporate Engagement, Monsanto Company, Creve Coeur, MO; 7 Regulatory Policy and Scientific Affairs, Bayer U.S. Crop Science Division, Chesterfield, MO

**Keywords:** food safety, toxicology, dietary risk assessment, glyphosate, pesticides

## Abstract

Understanding the magnitude and impact of dietary pesticide exposures is a concern for some consumers. However, the ability of consumers to obtain and understand state-of-the-science information about how pesticides are regulated and how dietary exposure limits are set can be limited by the complicated nature of the regulations coupled with an abundance of sources seeking to cast doubt on the reliability of those regulations. Indeed, these regulations are sometimes not well understood within health care professions. As such, the objective of this review is to provide a historical perspective as to how modern pesticides were developed, current trends in pesticide use and regulation, and measures taken to reduce the risk of pesticide use to the consumer. Throughout the review, we provide specific examples for some of the concepts as they apply to glyphosate—a pesticide commonly used by both farmers and consumers. In addition, we describe current efforts to monitor pesticide use. We are confident that this succinct, yet thorough, review of this topic will be of interest to myriad researchers, public health experts, and health practitioners as they help communicate information about making healthful and sustainable food choices to the public.

## Introduction

More than ever before, today's consumer exhibits a desire to understand where food comes from and how it was grown in a food milieu characterized by the convergence of agriculture, nutrition, and sustainability (1). Although previously separate topics, many health professionals (such as dietitians) are finding that conversations about food, agriculture, and nutrition have coalesced, especially as consumers make food-related decisions in a time of information abundance, and even information inundation leading to increased fear (2).

For instance, marketing food as “clean” or “free-from” is a recent trend (3) that consumers are utilizing (and demanding) to determine their desire to purchase a particular food. Interestingly, these designations and others like them appear to be more important to consumers for what is not in the food rather than what the food actually contains. This is important because, whereas there has historically been a focus on choosing a food based on its nutrients that might perform certain functions for human health, now purchasing trends seem to be focused more on choosing “simple” foods with fewer ingredients. Despite this trend, coupled with the finding that consumers view these claims to signal a healthier and less-processed food, “clean” and related terms are not indicative of macronutrient or micronutrient content nor do these terms bear US FDA definitions (4). As such, there is growing need to help educate the consumer as to what is and is not in foods, how to discern science-based food and nutrient claims from trendy and unregulated food-labeling trends, and what to monitor in terms of food composition.

One important application of this need centers around food labels and claims regarding pesticides commonly used in food production. Although food-related marketing abounds, most people are quite disconnected from the actual processes entailed in food production and farming. Consequently, the typical consumer may not understand the inputs farmers use and the decisions farmers face to produce food. For example, farmers analyze a variety of data to make as many as 40 decisions each growing season—decisions about the type of seed to plant, irrigation to utilize, pest control to apply, and much more. Without controlling for pests, farmers would lose an estimated 40% (5) of their yield, increasing the potential for expending further inputs, such as more land, water, seed, tractor fuel, labor hours, and pest-control products, to achieve necessary yields. In the meantime, Mintel reports that consumers describe healthy food as “natural” (63%) and pesticide-free (44%) (2), and the Natural Marketing Institute characterizes consumers as wanting more beneficial nutrients but wanting less of what allows farmers to produce adequate amounts of food (i.e., pesticides) (3).

From the moment farmers plant seeds in the ground, vulnerability to multiple pests, including weeds and insects, threatens the crop's potential. Therefore, protecting the seeds and plants by managing pests is one essential element of farming. Pest management takes into consideration the individual growing conditions and ecology of the field or farm to prevent pests from thwarting productivity, and at the same time, farmers’ decisions account for the health of the environment and humans as well as the economic impact (6). The dissonance present in what consumers prefer compared with the reasons farmers choose tools such as pesticides speaks to the need for explicating the role of crop protection.

This article reviews the process the US Environmental Protection Agency (EPA) uses to assess the safety of pesticides, determine safe exposure levels, establish allowable levels on food, and verify that these levels are not exceeded. Recent EPA reviews of the herbicide glyphosate provide specific examples of the steps in these processes. The article also discusses how the USDA and FDA monitor and report the levels of pesticides in the food supply and how that information informs the EPA's decisions.

## Development of Pesticides and Current Trends

The EPA defines the term “pesticide” as “Any substance or mixture of substances intended for (1) preventing, destroying, repelling, or mitigating any pest, (2) use as a plant regulator, defoliant, or desiccant, or (3) use as a nitrogen stabilizer” (7). Although this official definition is relatively recent, pesticides have been part of global agricultural production for millennia and have likely been long-essential for controlling insects, weeds, and diseases. For instance, there are reports of sulfur being used to control plant diseases dating back 4500 y to its first use in Sumeria. Mercury and arsenic salts were introduced later, and the insecticidal properties of chrysanthemum flower extracts (pyrethrum) were discovered ∼2000 y ago. Indeed, naturally occurring substances have long been leveraged to help control pests in agriculture (8).

Today many substances used in everyday life are registered as pesticides if they are marketed for their ability to mitigate a pest. Disinfecting agents are a common example. Products containing bleach and marketed for their ability to control pathogens must be registered with the EPA as a pesticide (9). Boric acid, often used as a whitening agent for washing clothes, is also registered as a pesticide for insect control (10). Vitamin D_3_ (cholecalciferol) is also a registered pesticide as well as an essential nutrient. When consumed in high doses, vitamin D_3_ can kill rats and mice, and products containing vitamin D_3_ that are intended for that purpose must be registered with the EPA as rodenticides (11).

In the early-to-middle 20th century as chemical synthesis became common, however, there was a shift toward the manufacture and use of pesticides with lower application rates, less toxicity to crop plants, and more specificity to their target organisms. Problems remained, however, particularly with many pesticides’ persistence in the environment and accumulation in the food chain, as occurred with organochlorine compounds such as dichloro-diphenyl-trichloroethane (DDT). For this and other reasons, since the early 1970s there has been greater emphasis on developing pesticides that degrade faster, do not accumulate, and have less toxicity to humans and wildlife. In fact, pesticide use in agriculture declined after the early 1980s, with insecticides showing the most reduction in the total weight applied. According to data compiled by the USDA’s Economic Research Service, the total weight of pesticides applied to the top 21 crops grown in the United States peaked in 1981 and, after a decline in the mid-1980s, has been steady over recent years (12). As such, it is important for those interested in this topic (and needing to communicate accurate information to the public) to recognize that today's pesticide-use landscape is much different from what it was even just a few decades ago.

Many of the pesticides used by today's farmers, in fact, contain the same active ingredients available to general consumers for home and garden uses. And, like consumers who use them at home, farmers have many options above and beyond pesticide application to control weeds, insects, and diseases. Indeed, pesticides often complement nonchemical methods to ensure the most-effective pest control (13). Nonetheless, when pesticides are used, it is important that all users—be they farmers or homeowners—choose the right product and apply it at the correct time and in the appropriate amount. Recent developments that improve precision in agriculture allow farmers to better control how much pesticide they apply and where it is applied. For instance, global positioning system (GPS)–directed sprayers allow targeted applications, increasing efficacy and decreasing waste. Variable-rate planting, fertilizer application, and weed control technologies also help reduce inputs and increase productivity (14). Indeed, farmers carefully plan and monitor their pesticide usage so as to apply as little as possible while reaping the desired benefit—namely, maximal agricultural production.

In addition to calculated application by farmers, and due to a long-standing interest in regulating pesticide use to optimize applicator and consumer health, the US federal government has enacted a series of dovetailing and complementary pieces of legislation over the last few decades. Understanding the purposes of these legislative documents is important to understanding the coordinated oversight and monitoring of pesticide use in US agriculture. Some of the most important of these regulatory elements are described in the next sections.

## The Federal Insecticide, Fungicide, and Rodenticide Act

One of the first such laws related to pesticide use was passed by the US Congress in 1947. This was the Federal Insecticide, Fungicide, and Rodenticide Act (FIFRA) aimed at controlling pesticide quality, and responsibility for overseeing the Act was given to the USDA. In time, regulations were strengthened to ensure that more was known about each chemical before it could be put on the market, and limitations were placed on how much of each active ingredient could remain in or on food products grown with each pesticide product. For instance, in 1972, FIFRA was amended to place a greater emphasis on pesticide safety and give the newly formed EPA authority over pesticide regulation. Today, FIFRA classifies all insecticides, herbicides, fungicides, rodenticides, and plant growth regulators as “pesticides” (7). In addition to synthetic pesticides, this classification also includes pesticides listed in the National Organic Program's approved substances list (15).

Today, the EPA initiates the review of a new pesticide when the entity that produces the pesticide, referred to as the “registrant,” submits the pesticide for registration under FIFRA. Registrants are responsible for conducting studies on the pesticide according to standardized study protocols based on global standards, and any adverse effects must be reported to the EPA. The EPA can also request additional data if the submitted studies do not completely enable an assessment of the proposed uses of the pesticide.

In addition to the initial review conducted to allow registration of a pesticide, the EPA also requires a registration review for all pesticides every 15 y. These registration reviews allow the agency to assess any new data that have become available since the last review, issue requirements for new data, and ensure that the information available for each pesticide meets current requirements. In addition, the EPA can take immediate action to restrict uses of a pesticide if pertinent new information becomes available, regardless of registration review status (16).

## The Federal Food, Drug, and Cosmetic Act

Alongside its responsibilities under FIFRA, the EPA also regulates pesticides under the Federal Food, Drug, and Cosmetic Act (FFDCA). Whereas FIFRA addresses approved uses, rates of usage, and environmental impacts, FFDCA addresses human exposures to pesticides and how the EPA sets allowable limits for pesticides in and on food (17). Before it can establish allowable limits on pesticide exposures—known as tolerances—the EPA must first conduct a risk assessment to determine acceptable levels. The following sections describe this process.

## The Food Quality Protection Act

The 1996 Food Quality Protection Act (FQPA) amended FFDCA to increase protections for human health, particularly for at-risk and sensitive subpopulations. Notably, FQPA requires that the EPA may only approve a tolerance if the agency can conclude “there is a reasonable certainty that no harm will result from aggregate exposure to the pesticide chemical residue, including all anticipated dietary exposures and all other exposures for which there is reliable information” (7).

In addition to creating this standard for approving pesticide tolerances, FQPA also requires cumulative exposure assessments for classes of chemicals with common modes of action. Before FQPA, each pesticide was assessed independently. Combining pesticides within each class ensures that tolerances account for multiple chemicals simultaneously, and that the cumulative risk still allows the EPA to reach a conclusion that there is a reasonable certainty that human exposures will not result in harm. In addition, FQPA requires the EPA's tolerances to consider all nonoccupational exposures across the diet, drinking water, and residential uses. This is important because, before FQPA, tolerances were required to consider only dietary exposures (7).

Finally, FQPA added specific considerations for infants and children by adjusting allowable exposures by a factor of 10 unless the EPA has data and information demonstrating that the allowable exposure for adults is adequately protective for the entire population (18). As such, these vulnerable populations are protected.

## EPA Data Requirements for Determining Human Health Risks

The EPA has standard toxicologic tests that must be submitted for the agency to conduct human health risk assessments. Test durations are acute, subchronic, and chronic. Acute toxicity testing involves short-term tests with a single exposure. These include oral, dermal, and inhalation exposures and endpoints such as frank toxicity, eye irritation, skin irritation, skin sensitization, and neurotoxicity. Subchronic testing involves intermediate-length exposures to multiple doses for periods ranging from 30 to 90 d. As with acute studies, exposure routes may be oral, dermal, and inhalation. Subchronic testing informs assessments of a pesticide's potential to adversely affect organ systems. Chronic testing considers long-term exposures to consecutive, repeated doses over most of the test animal's life span. Chronic testing evaluates the potential to cause damage to organs and organ systems and the potential to cause cancer. The EPA also requires a set of tests to assess the potential for a pesticide to damage DNA that also informs assessments of potential carcinogenicity (19).

In addition to the above tests, the EPA also requires studies to define how a pesticide is absorbed, distributed, metabolized, and excreted by mammals. These tests provide an understanding of whether a pesticide will accumulate in the body, be present in milk, or be metabolized into a form that presents unique safety concerns. This EPA-mandated testing also considers developmental and reproductive toxicity to evaluate the potential to cause fetal toxicity and birth defects (19). In 2009, the EPA also began requiring pesticides to undergo testing for their ability to mimic hormones or otherwise disrupt hormonal signaling. **Table 1** provides a partial list of studies that the EPA may consider when evaluating the safety of a new pesticide.

**TABLE 1:** Partial list of laboratory tests required on pesticides by the EPA^1^

Tests to establish basic properties
Physical property studies (melting point, flash point, solubility, vapor pressure, etc.)
Validated analytic methods
Acute (short-term) oral toxicity studies on rodents or nonrodents
Acute dermal toxicity studies on rodents or nonrodents
Acute inhalation toxicity studies on rodents or nonrodents
Acute eye irritation studies on rodents or nonrodents
Acute dermal irritation studies on rodents or nonrodents
Dermal sensitization studies on rodents or nonrodents
Tests to inform human health risk assessments (cancer and noncancer health effects)
90-d oral toxicity studies on rodents or nonrodents
90-d dermal toxicity studies on rodents or nonrodents
90-d inhalation toxicity studies on rodents or nonrodents
Chronic (long-term) feeding studies on rodents and nonrodents
Teratogenicity (birth defects) studies on 2 species
Two-generation mutagenicity (chromosome defects) tests on rodents
Gene mutation tests (in vitro)
Chromosomal aberration tests (in vitro)
Endocrine disruptor screening tests (in vivo and in vitro)
Neurotoxicity studies (in vivo)
Immunotoxicity studies (in vivo)
Other tests to inform environmental risk assessments
General metabolism (breakdown) studies of the product in plants and animals
Toxicity testing with metabolites as needed
Ecologic effects testing on nontarget plants and animals
Environmental fate tests (degradation in soil and water)

^1^Adapted from reference 19. EPA, Environmental Protection Agency.

For such studies to inform the EPA's safety assessment, the studies must meet quality requirements that allow the agency to understand exactly what substance was tested and how the data were collected, enabling the study to be reproduced if necessary. These data-quality requirements, referred to as “Good Laboratory Practices” (40 Code of Federal Regulations, Part 160), improved the reliability of safety studies and are also relied on by the FDA as well as regulatory agencies around the world. In concert with Good Laboratory Practices, the Organization for Economic Cooperation and Development (OECD) produced a series of guidelines for chemical testing to standardize study protocols for all OECD member countries. The OECD is a group of countries, including the United States, that develops solutions to common problems to promote harmonization in areas including regulatory requirements. The OECD's guidelines are harmonized with those of the EPA. Table 1 presents examples of the types of data the EPA requires to support its assessment.

**Text Box 1** presents the EPA's conclusions, taking an example with the herbicide glyphosate.

**Text Box 1 The EPA's Assessment of Human Health Safety Studies for Glyphosate**

A study of glyphosate's absorption, distribution, metabolism, and excretion in rats demonstrated that approximately one-third of an administered dose is absorbed, whereas the rest is excreted through feces largely as unmetabolized glyphosate. The absorbed portion of the dose was excreted through the urine. Small amounts of a single metabolite were present in urine and feces. Less than 1% of the absorbed dose remained in the rats, indicating that glyphosate does not accumulate in the body.

 After its review of appropriate toxicologic endpoints attributable to a single exposure (dose), including maternal toxicity in developmental toxicity studies conducted with glyphosate, the EPA concluded no acute reference dose (RfD) was necessary (20).

 No toxicity concerns were noted relevant to dermal or inhalation exposure endpoints. For subchronic and chronic toxicity, the EPA identified maternal toxicity in rabbits at an oral dosage of 350 mg · kg^–1^ · d^–1^ and selected 100 mg · kg body weight^–1^ · d^–1^ (mg · kg^–1^ · d^–1^) glyphosate as the no observed adverse effect level (NOAEL) to be used in the human health risk assessment (20).

 The EPA reviewed 9 chronic studies with rats and 6 chronic studies with mice and concluded that the available data support a conclusion that glyphosate is not likely to be carcinogenic (21).

 After reviewing the available studies, the EPA concluded that there was no evidence of reproductive or developmental toxicity. Likewise, they concluded that there was no evidence of neurotoxicity in any of the glyphosate safety studies (21).

 The EPA reviewed 56 in vitro studies and 28 in vivo studies of glyphosate's potential to damage DNA and concluded that glyphosate is not genotoxic and does not otherwise damage DNA (21).

 The EPA reviewed 10 EPA-mandated studies as well as data from the scientific literature to assess the potential for glyphosate to alter function of the endocrine system. They concluded that “glyphosate demonstrates no convincing evidence of potential interaction with the estrogen, androgen or thyroid pathways in mammals or wildlife” (22).

## Establishing an NOAEL and RfD

In addition to reviewing the types of studies described above, the EPA examines the body of toxicologic data to identify an NOAEL for each pesticide. An NOAEL is defined as the highest dose examined in all collective toxicologic studies to date that produced no detectable adverse effect on test animals (23). To translate the NOAEL from the animal toxicity studies to a numeric value protective of human health, the EPA applies a safety factor to the NOAEL. Typically, a value of 10 is used to account for extrapolations from test animals to humans and another safety factor of 10 is used to account for sensitive subpopulations to give a total safety factor of 100 (10 × 10). In other words, the NOAEL is divided by 100 and the resulting value is referred to as the RfD for the compound of interest (23). The EPA can also include additional safety factors (typically 3- to 10-fold) to account for especially sensitive subpopulations (e.g., infants, children, immunocompromised people) if the data warrant additional protection. Such an adjustment may result in dividing the NOAEL by 3 safety margins or as much as 1000 (10 × 10 × 10). RfD values, which are reported in units of milligrams of pesticide per kilogram of body weight per day (mg · kg^–1^ · d^–1^), can be interpreted as the amount of a particular compound a person may consume every day without raising concerns for adverse effects. The EPA requires that the sum of all exposures to a pesticide or class of pesticides across all approved uses cannot exceed the RfD. **Text Box 2** provides an example of how the NOAEL and RfD were determined for glyphosate.

**Text Box 2 EPA-Determined NOAEL and RfD Values for Glyphosate**

As described in Text Box 1, an acute RfD was not established based on the absence of an appropriate toxicologic endpoint attributable to a single dose. The EPA identified a lowest NOAEL of 100 mg · kg^–1^ · d^–1^ across all the major repeat-dose toxicity studies to calculate the chronic RfD (cRfD). The EPA divided the NOAEL by a factor of 10 to account for extrapolating results from an animal study to humans. The EPA divided the resulting value by another factor of 10 to account for sensitive subpopulations. These adjustments resulted in a cRfD of 1.00 mg · kg^–1^ · d^–1^ (21).

## Establishing Tolerances for Crops and Commodities

Once the RfD is calculated, the EPA sets tolerances for individual crops or groups of crops. A tolerance is defined as the legal limit for the level of a given pesticide in each crop or group of crops and is determined by measuring residues of the pesticide in question on a specific crop when the pesticide is applied according to label directions (24). Tolerances thus are not stand-alone safety standards (25). The EPA considers 3 types of data when setting tolerances.

The first type of data provides information regarding the toxicity of the pesticide and its breakdown products. The toxicity data are the same as those that were used to establish the NOAEL and RfD. The second type of data is the amount of pesticide remaining on each part of the crop after application at the maximum proposed usage rate (24). In the case of residues in animal products, the EPA requires data from animal feeding studies to determine the amount of a pesticide that could be present in muscle, milk, eggs, etc. The third type of data is the amount of pesticide that could be present in drinking water and the potential for exposures through residential or recreational exposure (24).

Commodities with pesticide residues greater than the allowed tolerance are “adulterated” and cannot be sold. In addition, if processing increases the amount of a pesticide in any processed product from the commodity to a level greater than the tolerance for the crop as a whole, the processed product is considered adulterated. In these cases, an additional tolerance for the pesticide on the processed product is required for the processed product to be sold (26).

**Table 2** presents the categories of data the EPA considers when setting tolerances. **Text Box 3** provides details of the tolerances established for glyphosate. All new tolerance values must be considered in the context of existing tolerances and be compared to the NOAEL and RfD through a dietary risk assessment, as described next.

**TABLE 2:** Data used to calculate pesticide tolerances^1^

Health effects	Dietary exposures	Other exposures
Effects of acute dietary exposures in animals	Quantity of residues when pesticide is used at maximum use rate under field conditions	Quantity of pesticide in drinking water based on environmental fate and persistence data or direct measurements
Effects of chronic dietary exposure in animals	Quantity of residues after processing of food (e.g., peeling, milling)	Quantity of oral and dermal pesticide exposure from exposure to treated soil, vegetation, and household residues (e.g., dust)
Effects of short-term (1–30 d) and intermediate-term (1–6 mo) oral, dermal, and inhalation exposure in animals	Quantity of residues in livestock after dietary exposure	
Cancer effects in animals (oral, dermal, inhalation)	Food supply monitoring data	

^1^Adapted from reference 24.

**Text Box 3 EPA-Approved Tolerances for Glyphosate**

The EPA has approved 158 tolerances for glyphosate on various crops and commodities. These tolerances reflect either direct use of glyphosate on the crop or incidental presence because of glyphosate use during the season.

 The maximum tolerance is 400 parts per million (ppm) for nongrass animal feed and the lowest is 0.05 ppm for eggs. Sixty-nine percent of the tolerances are ≤1 ppm. A list of the approved tolerances is published in 40 Code of Federal Regulations 180.364 (27).

## Dietary Risk Assessments for Pesticides

Requests for new tolerances are considered in the context of existing tolerances. To do this, the EPA considers all approved uses of a pesticide to calculate possible exposures to ensure the new tolerance still allows a conclusion of safety. Both acute (short-term) and chronic (long-term) exposures are considered. Acute exposures cover single exposures or exposures lasting a single day. Chronic exposures consider lifetime exposures and rely on food-consumption data, typically obtained from the USDA's NHANES What We Eat in America survey (28). The EPA uses these residue data to make conservative (i.e., worst-case) estimates of likely exposures for adults, children, and infants (29). The purpose of these estimates is not to provide an exact value for human exposure but rather to provide an upper-limit end estimate of exposures that likely overestimates actual exposures and ensures a protective approach to human health risk assessment.

To accomplish this task, food-consumption data are combined with data on anticipated residues, anticipated drinking water levels, and anticipated residential exposures. Data related to anticipated residues may be obtained from residue studies and information about the percentage of the crop that will be treated. The EPA can also make conservative assumptions that the pesticide will always be present at the tolerance level on 100% of the crop in question. Possible drinking water exposures are calculated with the use of validated models that take the physical properties of the pesticide and conservative assumptions about use rates and environmental variables such as soil type and precipitation (29). Residential exposures are estimated by examining other approved uses of the pesticide and determining whether contact with treated soil or airborne vapors are likely (30).

If the pesticide is part of a broader class of pesticides, the EPA considers cumulative exposures to pesticides with a common mechanism of toxicity. Through these cumulative risk assessments, the EPA considers whether the risks posed by a group of pesticides to adults as well as children meet the safety standard of “reasonable certainty of no harm” established by FQPA. After the exposure assessment, the EPA considers whether it is necessary to include additional safety factors to protect infants and children (7).

To make this determination, the agency reviews data on prenatal and postnatal effects from toxicologic studies. It also considers whether the database is complete with respect to prenatal and postnatal exposures. If data are sufficient and no effects were found, the EPA can rely on existing safety factors. If the data are not sufficient or if effects were found, the agency can add another safety factor, typically 10-fold (7). **Text Box 4** summarizes a recent dietary exposure assessment the EPA conducted for glyphosate.

**Text Box 4 EPA's Dietary Risk Assessment for Glyphosate**

The EPA considered human consumption of 507 commodities by 24 demographic groups in the United States to conduct a dietary risk assessment of glyphosate in 2016. These 507 commodities comprise all commodities and commodity groups with an approved tolerance for glyphosate residues. There are 158 approved tolerances for glyphosate on various commodities and commodity groups.

 To provide a conservative upper-end estimate of human exposures to glyphosate through the diet, the EPA assumed that 100% of all commodities with an approved tolerance had glyphosate present at a level equal to the tolerance. Commodity consumption data from NHANES/What We Eat in America were combined with tolerance values to produce an upper-end estimate of dietary glyphosate exposures in mg glyphosate · kg body weight^–1^ · d^–1^.

 The total US population had an estimated exposure of 0.091 mg · kg^–1^ · d^–1^. Adults over age 55 y had the lowest estimated exposure of 0.061 mg · kg^–1^ · d^–1^ and children ages 1–2 y had the highest estimated exposure of 0.23 mg · kg^–1^ · d^–1^. Dietary assessments for children typically have greater exposures on a body weight basis because they consume more food per unit of body weight.

 The NOAEL for glyphosate is 100 mg · kg^–1^ · d^–1^. This value is 1114 times greater than the estimated exposure for the total US population, 1632 times greater than the estimated exposure for adults over age 50 y, and 438 times greater than estimated exposures for children ages 1–2 y. FIFRA requires that exposures be ≥100-fold lower than the NOAEL; therefore, none of these exposure estimates raise a safety concern. The highest estimated exposure (0.23 mg · kg^ –1^ · d^–1^) is 23% of the cRfD (1.00 mg · kg^–1^ · d^–1^) (21).

 These estimated exposures are intended to provide a conservative upper-end estimate and greatly exceed values derived from both refined exposure assessments and direct measurements.

 Additional information on glyphosate exposure is available from the scientific literature.

 Acquavella et al. (31) used urine samples to calculate glyphosate exposures to farmers who use glyphosate and their family members. The maximum systemic dose for farmers was 0.004 mg/kg; for spouses, the maximum systemic dose was 0.00004 mg/kg; and for children, the maximum systemic dose was 0.0008 mg/kg.

 McGuire et al. (32) measured glyphosate in the urine of 41 breastfeeding women. Based on the measured levels, the fact that only 20% of dietary glyphosate is available, and that all available glyphosate is excreted through the urine, the authors reported that exposures were >4500-fold lower than the cRfD.

 Stephenson and Harris (33) conducted a refined exposure assessment that relied on measurements of glyphosate in various foods as well as information about the impact of processing on glyphosate residues. They reported that including these refinements reduced estimated glyphosate exposures by 67-fold.

## Monitoring Pesticides in the Food Supply

As part of approving a tolerance level, the EPA determines whether sufficient analytic methods are available to test for the presence of the pesticide in food. Both the USDA (34) and the FDA (35) have programs that rely, in part, on these testing methods to monitor pesticides on and in food. The FDA is responsible for enforcing tolerances. The USDA shares its findings with the FDA to determine whether any violations occurred.

In 2014, the Government Accountability Office criticized these testing programs because they emphasize testing for pesticides that pose the most risk rather than collecting samples to look for pesticides with the highest usage rates regardless of the risk that any detectable residue could pose (36). Both the FDA and USDA, however, assert that current testing strategies are robust and are successful at ensuring the safety of the US food supply (36). Indeed, the FDA's monitoring includes testing for >800 pesticides and includes those in use around the world, particularly those that do not have approved tolerances in the United States and may be present on imported foods. According to the FDA, the presence of a pesticide without an approved tolerance level accounts for >95% of the violations it discovers, and it is currently developing methods to expand the range of herbicides it can detect and quantify. This effort is expected to increase the number of analyzed pesticides to >1000 (36).

The USDA relies on the Pesticide Data Program within the Agricultural Marketing Service as well as the Food Safety Inspection Service (FSIS) to collect data. Information from the Pesticide Data Program allows the EPA to conduct dietary risk assessments required by FQPA, particularly for infants and children. The FSIS provides data for enforcement purposes and is intended to protect public health. The FSIS requires products that are being tested to be withheld from commerce until analytic data become available. Violations can result in recalls or other enforcement action (36). **Text Box 5** provides an overview of glyphosate monitoring in the United States, Canada, and the European Union.

**Text Box 5 Monitoring Data for Glyphosate in Food**

The USDA analyzed 300 samples of soybeans and found glyphosate on 271 (90.3%). The maximum level was 18.525 ppm (below the 20 ppm tolerance) and the minimum was 0.265 ppm, just above the detection limit of 0.25 ppm (34).

 The FDA collected 300 samples each of corn and soybeans and 120 samples each of milk and eggs as part of a special project to analyze glyphosate residues in 2016. Twenty-three percent of the corn samples and 34% of the soybean samples had detectable residues. Glyphosate was below the tolerance level for all samples. Glyphosate was not detected in the milk and egg samples (37).

 Canada reported glyphosate data for 3188 samples of vegetables, fruits, grains, and infant foods in 2016. Glyphosate was detectable in 29.7% of the samples and 1.3% of the samples had glyphosate levels above the maximum residue level (similar to a tolerance). Health Canada reviewed the data and concluded there was no human health concern (38).

 The European Food Safety Authority analyzed 5329 samples of vegetables, fruits, nuts, cereals, infant foods, and some animal products for glyphosate residues in 2015. Wheat was the most heavily sampled crop. Glyphosate was present in 3.1% of the samples and 0.09% of samples had glyphosate present at levels greater than the maximum residue level. In the sample with the highest concentration, the level of glyphosate was equivalent to 8% of the acceptable daily intake on an acute basis. For chronic exposures, European Food Safety Authority determined that glyphosate was present in the sampled items at a level equivalent to 0.05–0.16% of the acceptable daily intake (39).

The FDA's and USDA's monitoring efforts overlap several food categories. Both collect data on grains, dairy and eggs, fish, fruits, and vegetables from domestic and imported sources. The USDA has primary authority for monitoring pesticides in meat. The FDA is responsible for enforcing tolerances under FFDCA and it uses data from its own monitoring program as a primary source of information. The USDA reports any presumptive tolerance violations it finds to the FDA for review.

**Table 3** presents the FDA's most recent monitoring results for domestic cereals, eggs, fish, fruits, and vegetables (40) as well as the USDA's most recent results for meat (41). The data show that the majority of domestic grain (75.0%), dairy and egg (97.4%), fish (89.4%), and meat (99.77%) samples contained no detectable pesticide residues. Fruits and vegetables had 18.3% and 38.0% of samples without detectable residues, respectively. None of the domestic cereal, dairy and egg, or fish samples contained violative residues, whereas a small amount of fruit (2.2%), vegetable (3.8%), and meat (0.13%) samples did. The FDA's results show that violative residues were more common among imported commodities (40). As discussed previously, most of the violative residue findings are for pesticides that do not have an approved tolerance for the commodity in question rather than a violation of an existing tolerance (36).

**TABLE 3:** USDA Pesticide Data Program results across food categories^1^

Food category	Samples	Percentage of samples with no detectable residues	Percentage of samples with residues > tolerance
Grains	32	75.0	0
Dairy and egg	38	97.4	0
Fish	47	89.4	0
Fruits	224	18.3	2.2
Vegetables	266	38.0	3.8
Meat	3012	99.77	0.13

1 Data for food categories other than meat were obtained from the FDA (40). Data for meat were obtained from the USDA (41).

## Conclusions

In conclusion, pesticides—along with other nonchemical options—represent an effective and efficient means to control pests in food production, be it conventional or organic in terms of approaches. Advances in agricultural practices have, in fact, kept the total use of pesticides relatively unchanged since the mid-1980s. New pesticidal compounds undergo substantial safety testing and assessment by manufacturers before the data are reviewed by the EPA, and its risk assessments identify the amounts that may be consumed by both adults and children without raising concerns of adverse health impacts. The EPA also sets tolerances on a crop-by-crop basis to ensure that aggregate exposures do not exceed acceptable levels. Both the FDA and USDA monitor pesticides in the US food supply to ensure any pesticides present do not violate tolerances approved by the EPA. The vast majority of the violations the FDA and USDA detect are for pesticides that lack an approved tolerance on a specific commodity rather than an exceedance of an approved tolerance stemming from misapplication (36).

There is little doubt that this area of intense public concern and scrutiny is considered seriously by both food producers and federal regulatory agencies, and an understanding of the facts around pesticide usage and regulation will help the consumer make wise choices related to their food purchases. After all, understanding and communicating the interconnected balance among *1*) optimizing agricultural pesticide practices for more effective and efficient food production, *2*) benefits of these practices to nutrient and food availability around the globe, and *3*) potential risks posed by pesticide usage require solid, evidence-based knowledge of all these topics.

## Notes

The authors reported no funding received for this study.

Author disclosures: MKM, no conflicts of interest. WRR, MS, and JLV are employed by Bayer US Crop Science Division, a manufacturer of glyphosate and glyphosate-based herbicides.

Abbreviations used: cRfD, chronic reference dose; EPA, Environmental Protection Agency; FFDCA, Federal Food, Drug, and Cosmetic Act; FIFRA, Federal Insecticide, Fungicide, and Rodenticide Act; FQPA, Food Quality Protection Act; FSIS, Food Safety Inspection Service; NOAEL, no observed adverse effect level; OECD, Organization for Economic Cooperation and Development; ppm, parts per million; RfD, reference dose.
